# Supplementary material for: Anti-HMGB1 Antibody Therapy Ameliorates Spinal Cord Ischemia–Reperfusion Injury in Rabbits
Source: Int J Mol Sci. 2025 Sep 5;26(17):8643. doi: 10.3390/ijms26178643 (PMC12429464; doi:10.3390/ijms26178643)
Supplement: Supplementary file 1 [file ijms-26-08643-s001.zip › ijms-3815924-supplementary.pdf]

Supplemental Figure S1. Real-time PCR results when outliers are excluded from Figure 10

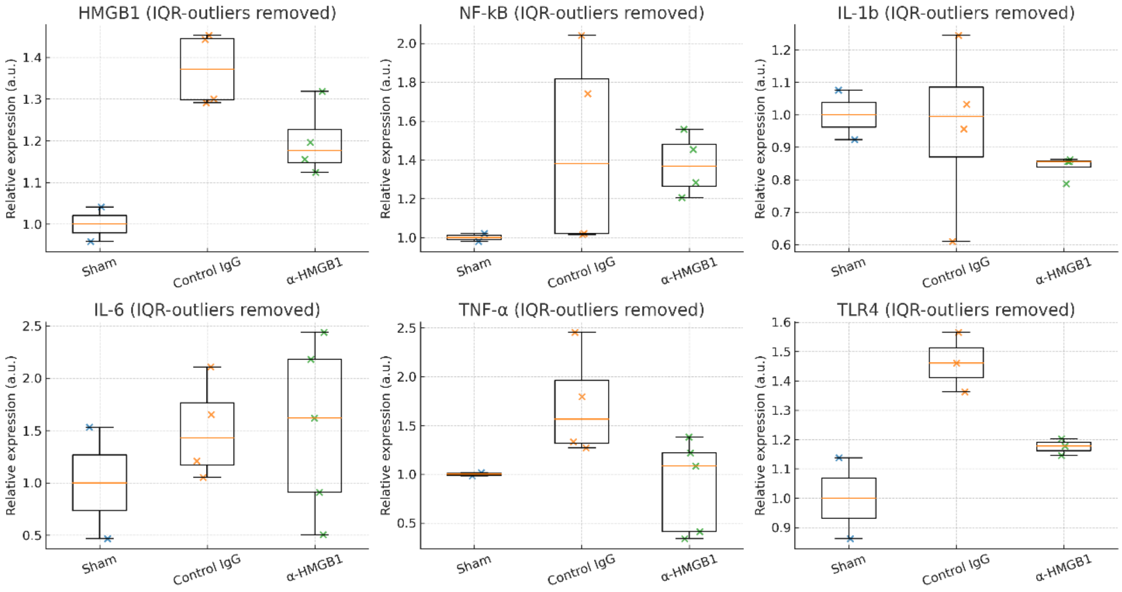

Supplementary Tables

Table S1. Sense and antisense primers used for the analysis of mRNA expression.

**Gene**	**Sense primer**	**Antisense primer**
HMGB1	5′-AAGGGAGTCGTCAAGGCTGA-3′	5′-CATCTTCCTCGTCTTCCTCTTCC-3′
TLR4	5′-GAGGAGGCTGTTGGTGGAAG-3′	5′-CTGAATACTGACACGCTAATGATGG-3′
NF-κB	5′-AGAGTTCACATCTGATGAGTTAATGG-3′	5′-TCTGGATCGGGAATTTCTAGCAAT-3′
IL-1β	5′-CTGTACCTGTCCTGCGTGATG-3′	5′GCTTGTCCTTGATTTCTATCTTGTTG-3′
IL-6	5′-CCTGAGGCCAAAGGTCAAGAAT-3′	5′-ATGAAGTGGATCGTGGTCGTC-3′
TNF-α	5′-CCTCATCTACTCCCAGGTTCTCT-3′	GAGGAGGTTGACCTTGTTCGG-3′
GAPDH	5′-TGAAGGTCGGAGTGAACGGATT-3′	5′-ATGTAGTGGAGGTCAATGAATGGAT-3′

Abbreviations: GAPDH, glyceraldehyde-3-phosphate dehydrogenase. HMGB1, High-mobility group box-1; IL-1β, Interleukin-1β; IL-6, Interleukin-6; NF-κB, Nuclear factor kappa-light-chain-enhancer of activated B cells; TLR4, Toll-like receptor 4; TNF-α, Tissue necrosis factor-α.

Table S2. Modified Tarlov score of each rabbit

	rabbit	6h	24h	48h
Sham	1	4	4	4
	2	4	4	4
HMGB1	1	4	4	4
	2	4	3	2
	3	4	4	2
	4	4	4	2
	5	4	4	3
control IgG	1	1	3	2
	2	4	1	1
	3	3	2	1
	4	4	4	2
	5	3	2	1

Table S3: Values from which the figures 1-9 are based.

Figure 2: Serum levels of HMGB1 (ng/ml)<br>Sham: n=2, Control IgG: n=5, α-HMGB1: n=5		P-value
Sham (pre)	4.3 ± 0.32 (4.0-4.7)	N/A
Sham (post)	4.4 ± 0.87 (3.5-5.2)	N/A
Sham (sacrifice)	5.8 ± 0.51 (5.3-6.3)	N/A
Control IgG (pre)	5.1 ± 0.31 (3.8-5.6)	vs Sham(pre): P=0.31
Control IgG (post)	6.0 ± 034 (5.3-7.2)	vs Sham (post): P=0.07
Control IgG (sacrifice)	9.8 ± 2.1 (6.4-18.3)	vs Sham (sacrifice): P=0.07
α-HMGB1 (pre)	5.1 ± 0.27 (4.5-6.1)	vs Control (pre): P=0.76
α-HMGB1 (post)	5.8 ± 0.22 (5.3-6.4)	vs Control (post): P=0.60
α-HMGB1 (sacrifice)	8.1 ± 0.83 (5.9-10.3)	vs Control (sacrifice): P=0.48
Figure 4-A : Viable neuron analysis in the anterior horns at three spinal cord levels per animal, 48 hours after ischemia-reperfusion injury (L7)<br>Sham: n=1, Control IgG: n=5, α-HMGB1: n=5		P-value

Sham	78	N/A
Control	7,1,7,16,1	N/A
α-HMGB1	78,14,14,17,18	vs Control: P=0.126
Figure 4-B & 4C: Viable neuron analysis in the anterior horns at three spinal cord levels per animal, 48 hours after ischemia-reperfusion injury<br>Sham: n=1, Control IgG: n=5, α-HMGB1: n=5		P-value
Sham	L5: 66, L6: 72, L7 78	N/A
Control	L5: 19,38,12,40,25<br>L6: 10,3,6,66,12<br>L7: 7,1,7,16,1	N/A
α-HMGB1	L5: 64,14,40,24,68<br>L6:73;8;30;454<br>L7: 78;14;14;17;18	vs Control: P=0.045
Figure 5B: Area of HMGB1-positive cells (μm^2)<br>Sham: n=2, Control IgG: n=5, α-HMGB1: n=5		P-value
Sham	7041 ± 294 (6625-7456)	N/A
Control	3336 ± 410 (2380-4583)	N/A

α-HMGB1	5399 ± 681 (3354-7529)	vs Control: P=0.031
Figure 5C: Activated microglia (counts/field)<br><br>Sham: n=2, Control IgG: n=5, α-HMGB1: n=5		P-value
Sham	3.5 ± 0.4 (3-4)	N/A
Control	48 ± 8.3 (25-69)	N/A
α-HMGB1	13 ± 3.1 (5-24)	vs Control: P=0.004
Figure 6: Myeloperoxidase-positive neutrophiles (counts/field)<br><br>Sham: n=2, Control IgG: n=5, α-HMGB1: n=5		P-value
Sham	6.5 ± 1.1 (5-8)	N/A
Control	17.6 ± 2.3 (12-27)	N/A
α-HMGB1	7.6 ± 0.8 (5-10)	vs Control: P=0.007
Figure 7A: Relative intensity of 4-HNE<br><br>Sham: n=1, Control IgG: n=5, α-HMGB1: n=5		P-value
Sham	1.72	N/A
Control	4.54 ± 0.84 (0.99-6.12)	N/A
α-HMGB1	2.07 ± 0.43 (1.04-3.88)	vs Control: P=0.047
Figure 7B: Relative intensity of cleaved caspase-3		P-value

Sham: n=1, Control IgG: n=5, α-HMGB1: n=5		
Sham	0.39	N/A
Control	0.84 ± 0.03 (0.74-0.94)	N/A
α-HMGB1	0.58 ± 0.03 (0.49-0.66)	vs Control: P=0.0005
		P-value
Sham	1.00 ± 0.03 (0.96-1.04)	N/A
Control	1.37 ± 0.04 (1.29-1.45)	N/A
α-HMGB1	1.20 ± 0.04 (1.12-1.32)	vs Control: P=0.030

Table 4S qPCR values for Figure 10

Marker	Sham n	Sham mean	Sham median	Sham min	Sham max	Control n	Control mean	Control median	Control min	Control max	α−HMGB1 n	α−HMGB1 mean	α−HMGB1 median	α−HMGB1 min	α−HMGB1 max	MW−U p (Control vs α−HMGB1)
HMGB1	2	1	1	0.958435	1.041565	5	1.751126307	1.442681363	1.291235989	3.268780935	5	1.302046639	1.196443852	1.124086217	1.715646941	0.222222222
NF−kB	2	1	1	0.979209	1.020791	5	1.904478076	1.740724762	1.013740297	3.705551064	5	1.534669212	1.453658219	1.205547174	2.172999897	1
IL−1b	2	1	1	0.923901	1.076099	5	2.176830193	1.032262949	0.609547484	7.041147264	5	0.773488411	0.85607584	0.505509642	0.862030319	0.095238095
IL−6	2	1	1	0.468207731	1.531792269	5	9.798558172	1.653153469	1.053522161	42.96889173	5	1.53075731	1.619132062	0.505303004	2.437193271	0.69047619
TNF−α	2	1	1	0.982673	1.017327	5	1.411522019	1.333098519	0.205154599	2.453401588	5	0.886347318	1.082812466	0.340277438	1.380110136	0.30952381
TLR4	2	1	1	0.862252	1.137748	5	1.903028212	1.460215434	1.054223848	4.073252455	5	1.155274064	1.177870746	0.810105234	1.440112216	0.150793651

Table 5S. qPCR effect size

Marker	Cliff's δ (all)	Cliff's δ 95% CI (all)	HL median diff (treated-control, all)	Cliff's δ (no outliers)	Cliff's δ 95% CI (no outliers)	HL median diff (treated-control, no outliers)
HMGB1	−0.52	[−1.000, 0.200]	−0.167149771	−0.75	[−1.000, 0.000]	−0.155839217
NF-kB	−0.04	[−1.000, 0.760]	−0.182732458	0	[−1.000, 1.000]	0.001011649
IL-1b	−0.68	[−1.000, −0.040]	−0.244512533	−0.5	[−1.000, 0.500]	−0.16948237
IL-6	−0.2	[−0.840, 0.600]	−0.299370041	0	[−0.800, 0.800]	0.020141676
TNF-α	−0.44	[−1.000, 0.360]	−0.577767733	−0.8	[−1.000, −0.200]	−0.891220213
TLR4	−0.6	[−1.000, 0.120]	−0.282344687	−1	[−1.000, −1.000]	−0.282344687
